# Sublytic C5b‐9 induces proliferation of glomerular mesangial cells via ERK5/MZF1/RGC‐32 axis activated by FBXO28‐TRAF6 complex

**DOI:** 10.1111/jcmm.14473

**Published:** 2019-06-11

**Authors:** Tianyi Yu, Lulu Wang, Chenhui Zhao, Baomei Qian, Chunlei Yao, Fengxia He, Yufeng Zhu, Mengyuan Cai, Mei Li, Dan Zhao, Jing Zhang, Yingwei Wang, Wen Qiu

**Affiliations:** ^1^ Department of Immunology Nanjing Medical University Nanjing People’s Republic of China; ^2^ Department of Medicine First Affiliated Hospital of Nanjing Medical University Nanjing People’s Republic of China; ^3^ Clinical Medical Science of the First Clinical Medical College Nanjing Medical University Nanjing People’s Republic of China; ^4^ The Laboratory Center for Basic Medical Sciences Nanjing medical University Nanjing People’s Republic of China; ^5^ Key Laboratory of Human Functional Genomics of Jiangsu Province Nanjing medical University Nanjing People’s Republic of China

**Keywords:** extracellular matrix, FBXO28‐TRAF6 complex, glomerular mesangial cells, proliferation, sublytic C5b‐9, ubiquitination

## Abstract

Mesangioproliferative glomerulonephritis (MsPGN) is characterized by the proliferation of glomerular mesangial cells (GMCs) and accumulation of extracellular matrix (ECM), followed by glomerulosclerosis and renal failure of patients. Although our previous studies have demonstrated that sublytic C5b‐9 complex formed on the GMC membrane could trigger GMC proliferation and ECM expansion of rat Thy‐1 nephritis (Thy‐1N) as an animal model of MsPGN, their mechanisms are still not fully elucidated. In the present studies, we found that the levels of response gene to complement 32 (RGC‐32), myeloid zinc finger 1 (MZF1), phosphorylated extracellular signal‐regulated kinase 5 (phosphorylated ERK5, p‐ERK5), F‐box only protein 28 (FBXO28) and TNF receptor‐associated factor 6 (TRAF6) were all markedly up‐regulated both in the renal tissues of rats with Thy‐1N (in vivo) and in the GMCs upon sublytic C5b‐9 stimulation (in vitro). Further in vitro experiments revealed that up‐regulated FBXO28 and TRAF6 could form protein complex binding to ERK5 and enhance ERK5 K63‐ubiquitination and subsequent phosphorylation. Subsequently, ERK5 activation contributed to MZF1 expression and MZF1‐dependent RGC‐32 up‐regulation, finally resulting in GMC proliferative response. Furthermore, the MZF1‐binding element within RGC‐32 promoter and the functions of FBXO28 domains were identified. Additionally, knockdown of renal FBXO28, TRAF6, ERK5, MZF1 and RGC‐32 genes respectively markedly reduced GMC proliferation and ECM production in Thy‐1N rats. Together, these findings indicate that sublytic C5b‐9 induces GMC proliferative changes in rat Thy‐1N through ERK5/MZF1/RGC‐32 axis activated by the FBXO28‐TRAF6 complex, which might provide a new insight into MsPGN pathogenesis.

## 1 INTRODUCTION

Mesangioproliferative glomerulonephritis (MsPGN) is a kidney disease characterized by the proliferation of glomerular mesangial cells (GMCs) and accumulation of the extracellular matrix (ECM) that can cause glomerulosclerosis and renal failure in MsPGN patients.^1^, ^2^, ^3^ Although many experiments have proved that complement C5b‐9 complexes are deposited in the glomeruli of MsPGN patients,^4^, ^5^ the roles and mechanisms of C5b‐9 in GMC proliferation and ECM synthesis have not been fully illuminated.

Rat Thy‐1 nephritis (Thy‐1N) is a widely used animal model for studying MsPGN.^6^, ^7^, ^8^ The pathological changes of Thy‐1N include GMC apoptosis, GMC proliferation and ECM accumulation. It has been confirmed that rat Thy‐1N is complement‐dependent, particularly sublytic C5b‐9‐dependent.^9^, ^10^ Our early studies have disclosed that sublytic C5b‐9 stimulation in vitro can induce rat GMC proliferation and ECM secretion.^11^, ^12^ But, the underlying mechanism of sublytic C5b‐9‐triggered GMC proliferative pathological changes in Thy‐1N rats remains largely unknown.

It is well accepted that cell proliferation is related to the overexpression of some pro‐proliferative genes.^13^ Response gene to complement 32 (RGC‐32) was first identified in the oligodendrocytes stimulated with complement.^14^ Reportedly, RGC‐32 overexpression is correlated with the proliferation of endothelial cells, smooth muscle cells and colon cancer cells in response to a variety of stimuli including sublytic C5b‐9.^15^, ^16^, ^17^ At the initial stage of our experiments it was found that RGC‐32 expression was up‐regulated both in the renal tissues of Thy‐1N rats (in vivo) and in the GMCs stimulated with sublytic C5b‐9 (in vitro). However, the functions of RGC‐32 in sublytic C5b‐9‐mediated GMC proliferation and ECM secretion are unexplored.

As we know, gene expression is often regulated by special transcriptional factors.^18^ Myeloid zinc finger 1 (MZF1) is a transcription factor of many genes ie c‐Myc, connective tissue growth factor (CTGF), and MZF1 can obviously promote cell proliferation and ECM synthesis.^19^, ^20^, ^21^ In the initial stage of this study, we not only revealed a potential MZF1‐binding site in the promoter of RGC‐32 gene, but also proved that the MZF1 expression increased simultaneously with RGC‐32 both in vivo and in vitro. Therefore, the role of MZF1 and the relationship between MZF1 and RGC‐32 in GMC proliferation and ECM production upon sublytic C5b‐9 stimulation need to be clarified.

Generally, mitogen‐activated protein kinase (MAPK) signal pathways are involved in cellular proliferation and ECM production.^22^, ^23^ Extracellular signal‐regulated kinase 5 (ERK5)/MAPK7 is a proliferation‐related signalling molecule,^24^, ^25^ and its activation is associated with the expression of transcriptional factors eg c‐Jun and c‐Fos.^26^, ^27^ Given that our results showed that ERK5 phosphorylation was enhanced and the phase of ERK5 phosphorylation was earlier than that of MZF‐1 and RGC‐32 expression both in vivo and in vitro, whether ERK5 phosphorylation can regulate MZF1 and RGC‐32 expression as well as GMC proliferation and ECM expansion is required to be studied.

Protein ubiquitination is a cascade reaction through E1‐E3 ubiquitin ligases and the linkage of ubiquitin to adjacent ubiquitin can occur through lysine 48 (K48) or lysine 63 (K63). K48‐ubiquitination targets proteins to proteasome for degradation,^28^ whereas K63‐ubiquitination functions as scaffolds in signalling pathways, recruiting and activating downstream molecules.^29^ Reportedly, F‐box proteins are critical components of the SCF (Skp1‐CUL‐1‐F‐box protein) type E3 ubiquitin ligase complex and are involved in substrate recognition and recruitment for ubiquitination.^30^ Besides, TNF receptor‐associated factor 6 (TRAF6) also plays a vital role in intracellular signal transduction as an E3 ubiquitin ligase for K63‐ubiquitination.^31^ Our initial stage of the study showed that the expression of F‐box only protein 28 (FBXO28) and TRAF6 was up‐regulated, and FBXO28 and TRAF6 formed protein complex binding to ERK5. Additionally, our previous studies have demonstrated that TRAF6 can induce Akt1 K63‐ubiquitination in response to sublytic C5b‐9.^11^ Hence, we also want to know whether ERK5 phosphorylation can be regulated by FBXO28‐TRAF6 complex mediated by ERK5 K63‐ubiquitination, and what is the possible effect of ERK5 activation on MZF1 and RGC‐32 expression.

In the current research, we examined the levels of RGC‐32, MZF1, p‐ERK5, FBXO28 and TRAF6 both in vivo and in vitro and then determined the roles of these proteins in GMC proliferation and ECM secretion. Meanwhile, the regulatory effect of MZF1 on RGC‐32 gene transcription and the upstream regulatory mechanism of MZF1 and RGC‐32 expression (namely FBXO28‐TRAF6 complex‐dependent ERK5 K63‐ubiquitination and phosphorylation) were assessed in vitro. Moreover, the effects of silencing renal FBXO28, TRAF6, ERK5, MZF1 and RGC‐32 genes on GMC proliferative changes of Thy‐1N rats were also evaluated in vivo.

## 2 MATERIALS AND METHODS

### 2.1 Reagents

Monoclonal antibodies (Abs) against TRAF6 (1660‐1) and ERK5 (1719‐1) were supplied by Epitomics (Burlingame, CA, USA). Monoclonal Abs against cyclin D2 (3741), proliferating‐cell nuclear antigen (PCNA, 2586), K63‐linkage Specific Polyubiquitin (K63‐ubiquitin, 5621), His (2366 and 12698), HA (3724), Flag (14793) and β‐actin (3700) as well as polyclonal Abs against Phospho‐ERK5 at Thr218/Tyr220 (p‐ERK5, 3371) were purchased from Cell Signaling Technology (Danvers, MA, USA). Polyclonal Abs against specificity protein 1 (SP1, ab13370), collagen IV (ab6586) and MZF1 (BS5810) were supplied by Abcam (Cambridge, UK) and Bioworld Technology (Nanjing, China). Monoclonal Abs against FBXO28 (sc‐376851), TRAF6 (sc‐8409), p‐ERK5 (sc‐135760), MZF1 (sc‐293218), fibronectin (FN, ab6328) and polyclonal Abs against RGC‐32 (sc‐84222) were from Santa Cruz Biotechnology (Dallas, TX, USA). Biotin‐conjugated polyclonal Abs against RGC‐32 (bs‐9079R‐Biotin) were purchased from BiossInc (Woburn, MA, USA). HRP‐conjugated anti‐mouse IgG (ab6789) and HRP‐conjugated Streptavidin (ab7403) as well as HRP‐conjugated anti‐rabbit IgG (Conformation Specific, 5127) were supplied by Abcam and Cell Signaling Technology.

Rabbit polyclonal Ab against Thy‐1 antigen (Thy‐1 Ab) was prepared according to previously published procedures.^32^, ^33^ Pooled normal human sera (NHS) from 30 healthy adult donors were used as a source of complement and heat‐inactivated human serum (HIS) was obtained by incubating the NHS at 56°C for 30 minutes. Human C6‐deficient serum (C6DS) was obtained from Sigma‐Aldrich (St. Louis, MO, USA). Purified human complement C6 was from Sino Biological Inc (Beijing, China). Cell counting kit‐8 (CCK‐8) was purchased from Dojindo Laboratories (Kumamoto, Japan). Lipofectamine 2000 and pcDNA3.1 were from Invitrogen (Carlsbad, CA, USA). The expression plasmids of pEGFP‐N1 and pIRES2‐EGFP were supplied by Clontech (Mountain View, CA, USA). The luciferase reporters of pGL3‐Basic and pRL‐SV40 were purchased from Promega (Madison, WI, USA). The shRNA plasmids of pGPU6/GFP and GV102 were from GenePharma (Shanghai, China) and Gene Chem (Shanghai, China). An EZ‐ChIP^™ ^chromatin immunoprecipitation kit was from Millipore (Bedford, MA, USA).

### 2.2 Animals and cells

Male Sprague‐Dawley (SD) rats were purchased from B&K Universal Ltd. (Shanghai, China). All animal experiments were performed in compliance with the guide for the care and use of laboratory animals and were approved by the Institutional Animal Care and Use Committee of Nanjing Medical University. Rat GMCs and 293T cells were provided by China Centre for Type Culture Collection (Wuhan, China) or American Tissue Culture Collection (Manassas, VA, USA).

### 2.3 Thy‐1N induction

Normal male SD rats (180‐200 g) were given Thy‐1 Ab (0.75 ml/100 g) by a single i.p. injection to induce Thy‐1N. Normal rabbit sera (NRS, 0.75 ml/100 g, i.p.) were injected into rats as a control. The rat renal cortexes were obtained by kill at different time points and then examined for the expression and phosphorylation of corresponding proteins.

### 2.4 Cell culture and sublytic C5b‐9 stimulation

Rat GMCs were cultured in minimal essential medium (MEM) plus 10% foetal bovine serum (FBS) as previously described.^12^, ^34^ The selection of Thy‐1 Ab and the complement concentration used in this study was 5% Thy‐1 Ab and 4% NHS as previously reported.^9^, ^34^ For grouping experiments, GMCs were treated as follows: (a) 5% Thy‐1 Ab + 4% NHS, (b) 5% Thy‐1 Ab, (c) 5% Thy‐1 Ab + 4% HIS, (d) 5% Thy‐1 Ab + 4% C6DS, (e) MEM, (f) 5% Thy‐1 Ab + 4% C6DS + 2 mg/L C6, (g) 5% Thy‐1 Ab + 4% C6DS + PBS. Besides, 293T cells were maintained in Dulbecco's modified Eagle medium (DMEM) supplemented with 10% FBS.

### 2.5 Expression plasmid construction

The expression plasmids of pcDNA3.1/FBXO28‐His, pEGFP‐N1/ERK5‐His, pEGFP‐N1/ERK5‐Flag, pIRES2‐EGFP/MZF1‐Flag and pIRES2‐EGFP/RGC‐32‐His were constructed by inserting the coding sequences (CDS) of rat FBXO28, ERK5, MZF1 and RGC‐32 gene into pcDNA3.1, pEGFP‐N1 and pIRES2‐EGFP respectively. Briefly, the FBXO28 gene was synthesized directly, and ERK5, MZF1 and RGC‐32 genes were amplified using polymerase chain reaction (PCR) from the cDNA of rat GMCs. The synthesized DNA or PCR products and plasmids were digested with restriction enzymes (FBXO28: *Hind*Ⅲ and *EcoR*V; ERK5: *Xho* I and *BamH*I; MZF1 and RGC‐32: *Bgl* II and *Sal* I) and then ligated with T4 DNA ligase. pcDNA3.1/FBXO28∆1‐His (F‐box domain deletion) and pcDNA3.1/FBXO28∆2‐His (predicted coiled coil domain deletion) were constructed by General Biosystems (Chuzhou, China). The expression plasmids of pcDNA3.1/HA‐TRAF6^WT^ and pcDNA3.1/HA‐TRAF6^C70A^ (C70 → A70 mutant) were constructed in our previous study.^11^ The PCR primer sequences are listed in Table S1.

### 2.6 shRNA vector generation

To silence rat FBXO28, ERK5, MZF1 and RGC‐32 genes, different shRNA sequences were designed. The plasmids of shFBXO28, shERK5, shMZF1 and shRGC‐32 were constructed with pGPU6‐GFP (for FBXO28, ERK5 and RGC‐32) and GV102 (for MZF1) respectively. Meanwhile, the corresponding scrambled shRNA plasmids (shCTR) were produced as a negative control. The sequences of the above‐mentioned most effective shRNA are as follows: shFBXO28, AACAAGTTAAAGCACAACTAC; shERK5, GAACTGAAGATCCTCAAAC; shMZF1, GTCCAGAGGTACATTCCAA; shRGC‐32, CAAATTAGGTGACACTAAA; shCTR, TTCTCCGAACGTGTCACGT.

### 2.7 Cellular transfection

Plasmids were transfected into rat GMCs with Neon^™^ transfection system (Invitrogen) according to previously published procedures.^11^ Plasmids were transfected into 293T cells with Lipofectamine 2000 according to the manufacturer's instructions.^35^ The transfection efficiency was examined by the fluorescence of GFP (Figure S1).

### 2.8 CRISPR/Cas9‐mediated FBXO28 and TRAF6 gene knockout in rat GMCs

FBXO28‐ and TRAF6‐deficient rat GMCs cell lines were established using CRISPR/Cas9 respectively. Short guide RNAs (sgRNAs) targeting exon 1 of rat FBXO28 gene and TRAF6 gene were designed with CRISPR Design and constructed into pGK1.1 (Genloci Biotechnlogy, Nanjing, China). Sequences were as follows: FBXO28‐sgRNA: 5′‐AACAGGAGCCGCCGTCGCCGTGG‐3′; TRAF6‐sgRNA: 5′‐TGTGGAGTTTGACCCACCTTTGG‐3′. Different pGK1.1/TRAF6‐sgRNA and pGK1.1/FBXO28‐sgRNA were respectively transfected into rat GMCs with Neon^™^ transfection system. The positive clones were selected by DNA sequence. Finally, FBXO28 and TRAF6 gene knockout was further identified with IB analysis (Figure S2).

### 2.9 Immunoblotting (IB) analysis

The cultured GMCs and rat renal tissues were lysed using RIPA lysis buffer. Equal amounts (30 μg/lane) of protein were subjected to SDS‐PAGE gel. IB assay was performed as described before.^9^ Primary Abs against FBXO28 (sc‐376851), TRAF6 (1660‐1), ERK5 (1719‐1), p‐ERK5 (3371), MZF1 (BS5810), SP1 (ab13370), RGC‐32 (sc‐84222), cyclin D2 (3741), PCNA (2586), FN (ab6328), collagen IV (ab6586), His (12698), HA (3724), Flag (14793) and β‐actin (3700) as well as HRP‐conjugated anti‐mouse IgG (ab6789) and anti‐rabbit IgG (5127) were used to detect the expression of corresponding proteins. The bands were visualized using a regular X‐ray film through ECL detection system. The density of radiographic bands was analysed by using Quantity One (Bio‐Rad, Hercules, CA, USA).

### 2.10 Co‐immunoprecipitation (co‐IP)

Co‐IP was performed to pull down target protein complex as described previously.^36^ Briefly, the co‐IP experiment was done to pull down ERK5 protein complex with anti‐ERK5 (1719‐1) from the GMCs or anti‐His (2366) from 293T, then the IB assay was used to detect the levels of corresponding proteins in the co‐IP‐complex or in whole cell extract (WCE).

### 2.11 Luciferase reporter assay

The plasmid of pGL3/RGC‐32‐full‐length (pGL3/RGC‐32‐FL) was constructed by inserting rat RGC‐32 promoter (−1094 ~ +93 nt) into pGL3‐Basic vector through *Mlu* I and *Bgl* II restriction sites. We also amplified different truncated promoter fragments (−594 ~ +93, −394 ~ +93, −194 ~ +93 and +8 ~ +93 nt) using PCR and cloned them into the pGL3‐Basic. PCR primer sequences used here are listed in Table S2. pGL3/RGC‐32 mutant (GGTGGGGA‐GATAAAAA, pGL3/RGC‐32‐FL‐M) was constructed by General Biosystems (Chuzhou, China). The activity of the RGC‐32‐FL promoter and its different deletion fragments or mutant in GMCs or 293T cells was detected using luciferase reporter assay.^37^

### 2.12 Chromatin immunoprecipitation (ChIP)

ChIP was done by using an antibody against Flag (14793), and preimmune IgG respectively as mentioned previously.^37^ A proximal region in RGC‐32 promoter (−220 ~ −50 nt) was amplified from the immunoprecipitated chromatin by PCR using a pair of primers (sense, 5′‐CGGCGACACTAGTCG‐3′ and antisence, 5′‐CCCGCCAGCTACACCCTCTT‐3′).

### 2.13 Mass spectrometry

Anti‐ERK5 antibody (1719‐1) was used to perform co‐IP to pull down ERK5 protein complex from the GMCs stimulated with sublytic C5b‐9 for 5 hours and then 50 µg of protein was run in an SDS‐PAGE gel. Subsequently, the target protein was obtained again by cutting the gel after silver staining. Finally, mass spectrometry analysis was performed to scan the possible proteins binding to ERK5.

### 2.14 CCK‐8 assay

Rat GMCs after various treatments were incubated with CCK‐8 for the last 2 hours. The formazan product was visualized at an absorbance of 450 nm and the absorbance was directly proportional to the cell number.

### 2.15 Lentiviral shRNA packing and in vivo experiments

LV‐shFBXO28, LV‐shTRAF6, LV‐shERK5, LV‐shMZF1, LV‐shRGC‐32 and LV‐shCTR were provided by GenePharma (Shanghai, China). The shRNA oligonucleotide sequence against TRAF6 was GGTAAAGTGTCCAAATAAA.^11^ The shRNA oligonucleotide sequences to silence FBXO28, ERK5, MZF1 and RGC‐32 genes as well as control shRNA were the same as the above‐mentioned shRNA sequences used in vitro. To confirm the roles of these genes in the GMC proliferation and ECM secretion of Thy‐1N rats, male SD rats were divided into eight groups (n = 6 in each time‐point/group), namely, (a) NRS (0.75 ml/100 g, ip), (b) Thy‐1N (Thy‐1 Ab, 0.75 ml/100 g, ip), (c) LV‐shFBXO28 + Thy‐1N, (d) LV‐shTRAF6 + Thy‐1N, (e) LV‐shERK5 + Thy‐1N, (f) LV‐shMZF1 + Thy‐1N, (g) LV‐shRGC‐32 + Thy‐1N, (h) LV‐shCTR + Thy‐1N. Here, the rats allocated to 3‐8 groups were treated as described previously.^36^ The cortexes of rats were collected by sacrifice at 5 hours, 10 hours and 7 days after Thy‐1 Ab injection. GFP expression was observed to define the efficiency of lentivirus infection in the kidneys (Figure S3).

### 2.16 Proliferative change examination

For light microscopy (LM), the histological sections (4 μm) of rat renal cortex samples were stained with H Haematoxylin and eosin and the mean number of total glomerular cells in each rat was counted. The mean number of 100 glomerular cross‐sections from each rat was calculated and expressed. For electron microscopy (EM), ultrathin sections of rat renal cortex samples were stained with uranyl acetate and lead citrate and the ultrastructural changes were observed.^32^, ^38^

### 2.17 Immunohistochemistry (IHC) staining

Formalin‐fixed and paraffin‐embedded renal tissue sections of rats were incubated with primary antibodies against TRAF6 (sc‐8409), p‐ERK5 (sc‐135760), MZF1 (sc‐293218) and RGC‐32 (bs‐9079R‐Biotin) and then incubated with HRP‐conjugated anti‐mouse IgG (ab6789) or HRP‐conjugated Streptavidin (ab7403). DAB staining was performed. Quantitative analysis was performed using ImagePro Plus (Media Cybernetics, Inc Rockville, MD, USA) to determine the area of TRAF6, p‐ERK5 and RGC‐32 expression and the number of MZF1‐positive cells.

### 2.18 Urinary protein excretion

The 24 hours rat urine samples in different groups were collected from the sixth day to the seventh day after Thy‐1N induction. The contents of urinary protein (mg/24 h) of rats were measured using the total protein UC FS (DiaSys Diagnostic Systems, Holzheim, Germany).

### 2.19 Statistical analysis

Data are presented as means ± SD. *t* test or One‐way ANOVA followed by Bonferroni post‐hoc test was used to determine significant differences among groups. *P* < 0.05 was considered significant.

## 3 RESULTS

### 3.1 RGC‐32 expression is increased both in the renal tissues of Thy‐1N rats and in the GMCs stimulated with sublytic C5b‐9

**Figure 1:**
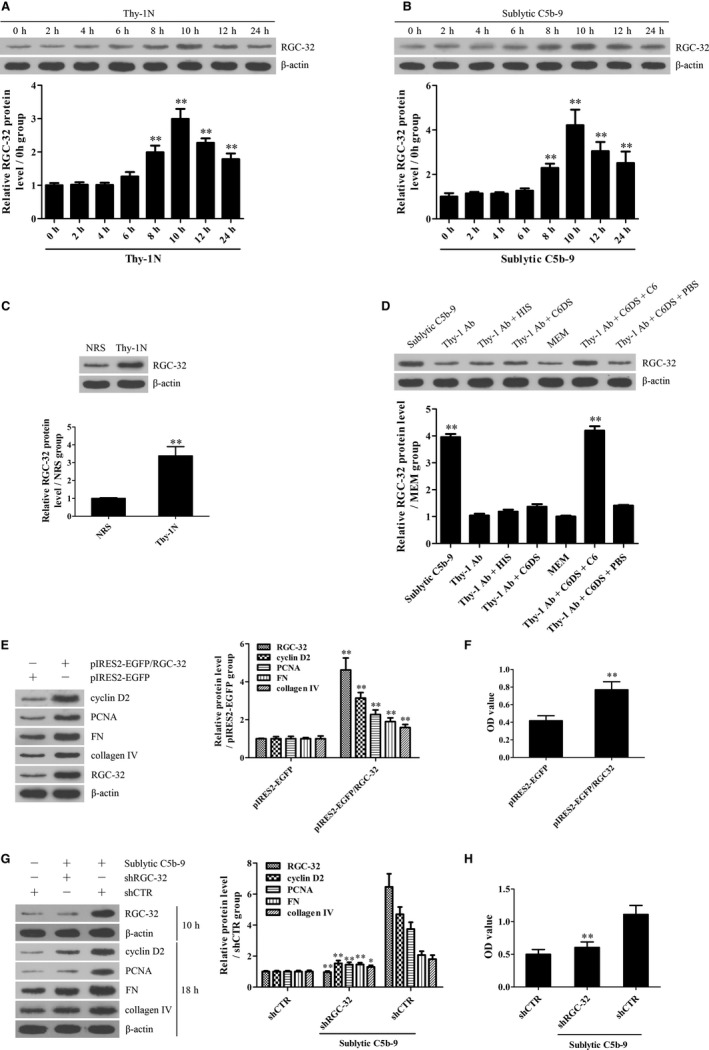
The expression and roles of RGC‐32 in GMC proliferation and ECM secretion exposed to sublytic C5b‐9. A and B, RGC‐32 protein in the renal tissues of Thy‐1N rats (A) and in the GMCs exposed to sublytic C5b‐9 (B) for different time‐points was examined using IB assay. **P < 0.01 vs 0 hour time‐point. C, RGC‐32 protein in the renal tissues of Thy‐1N and NRS rats at 10 hours was examined using IB analysis. **P < 0.01 vs NRS group. D, Rat GMCs were divided into different groups. At 10 hours after treatment, RGC‐32 protein was determined using IB experiments. **P < 0.01 vs other groups. E and F, Rat GMCs were transfected with pIRES2‐EGFP/RGC‐32 or pIRES2‐EGFP for 48 hours and 72 hours and then the protein levels of RGC‐32, cyclin D2, PCNA, FN and collagen IV in the GMCs were detected using IB assay (E, 48 hours) and cellular proliferation was also determined with CCK‐8 assay (F, 72 hours). **P < 0.01 vs pIRES2‐EGFP group. G and H, Rat GMCs were transfected with shRGC‐32 or shCTR for 48 hours followed by sublytic C5b‐9 treatment for different time‐points. The expression of RGC‐32 at 10 hours as well as cyclin D2, PCNA, FN and collagen IV at 18 hours were detected using IB assay (G). Additionally, GMC proliferation at 48 hours was determined with CCK‐8 assay (H). *P < 0.05, **P < 0.01 vs shCTR + sublytic C5b‐9 group. Results from one representative experiment out of three are shown. Data are represented as means ± SD (n = 6 in vivo, n = 3 in each group for IB in vitro, n = 5 in each group for CCK‐8)

RGC‐32 expression was examined in the renal tissues of Thy‐1N rats (in vivo) and in the GMCs exposed to sublytic C5b‐9 (in vitro). The experiments revealed that the protein levels of RGC‐32 were elevated in a time‐dependent manner, peaked at 10 hours both in vivo and in vitro (Figure 1A‐C). IHC staining further showed that RGC‐32 expression was enhanced mainly in the glomeruli of rats (Figure S4). To make sure that RGC‐32 up‐regulation was caused by the C5b‐9 assembly, rat GMCs were divided into different groups of sublytic C5b‐9 (Thy‐1 Ab + NHS), Thy‐1 Ab, Thy‐1 Ab + HIS, Thy‐1 Ab + C6DS, MEM, Thy‐1 Ab + C6DS + C6 and Thy‐1 Ab + C6DS + PBS respectively for 10 hours.^36^, ^39^ The results displayed that RGC‐32 expression was markedly increased in the sublytic C5b‐9 and Thy‐1 Ab + C6DS + C6 groups, but not in the other groups (Figure 1D). Here, adding C6 back to C6DS ie Thy‐1 Ab + C6DS + C6 group could recover the ability to induce RGC‐32 production in the GMCs, confirming that RGC‐32 expression is triggered by sublytic C5b‐9.

### 3.2 RGC‐32 induction is essential for GMC proliferation and ECM production induced by sublytic C5b‐9

To make sure whether RGC‐32 up‐regulation can promote GMC proliferation and ECM secretion, pIRES2‐EGFP/RGC‐32 was transfected into rat GMCs to overexpress RGC‐32 and then the levels of cyclin D2 and PCNA as two proliferative markers, as well as the levels of FN and collagen IV as two ECM molecules were detected. Additionally, GMC proliferation was also determined with the CCK‐8 assay. The results showed that RGC‐32 overexpression markedly enhanced cyclin D2, PCNA, FN and collagen IV expression as well as GMC proliferation (Figure 1E,F). Furthermore, RGC‐32 knockdown with shRGC‐32 could not only notably suppress cyclin D2, PCNA, FN and collagen IV expression, but also obviously inhibit GMC proliferation upon sublytic C5b‐9 (Figure 1G,H). Collectively, these data indicate that RGC‐32 induction is necessary for GMC proliferation and ECM secretion triggered by sublytic C5b‐9.

### 3.3 Identification of transcriptional factors for RGC‐32 gene promoter in response to sublytic C5b‐9

**Figure 2:**
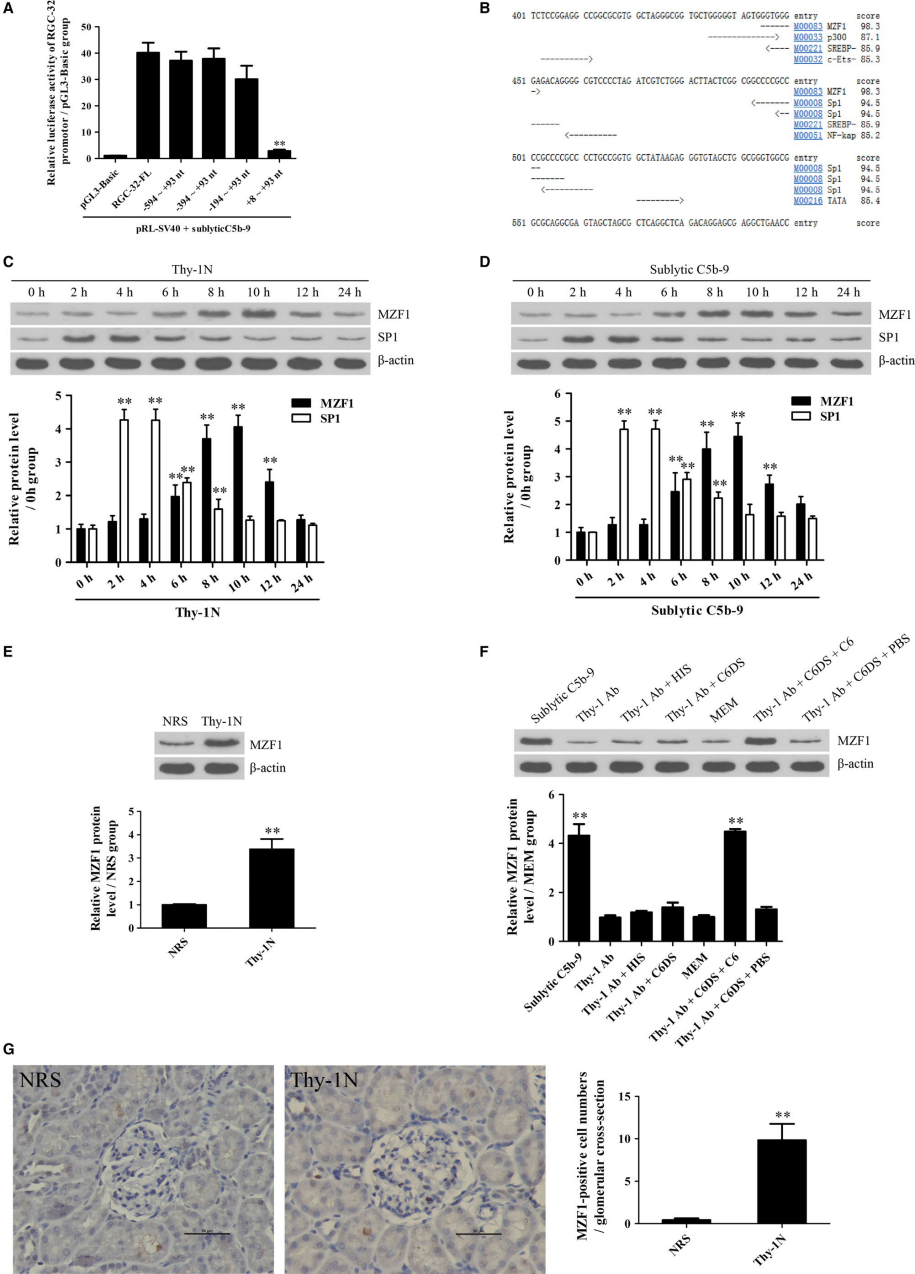
Analysis of RGC‐32 gene promoter and detection of potential transcriptional factors. A, Luciferase reporter plasmids of RGC‐32 gene promoter (FL or different deletion fragments) and pRL‐SV40 were co‐transfected into rat GMCs for 48 hours followed by sublytic C5b‐9 stimulation for 10 hours and then the luciferase activity was detected. **P < 0.01 vs FL group. B, Transcriptional factor binding elements in the −194 ~ +8 nt region of rat RGC‐32 gene promoter were predicted using TFsearch software. C and D, The protein levels of MZF1 and SP‐1 were detected using IB experiments both in the renal tissues of Thy‐1N rats (C) and in the GMCs induced by sublytic C5b‐9 (D). **P < 0.01 vs 0 hour time‐point. E and F, MZF1 protein in the renal tissues of Thy‐1N and NRS rats at 10 hours was examined using IB analysis (E) and IHC staining (F, Magnification, ×400). **P < 0.01 vs NRS group. G, Rat GMCs were divided into different groups. At 10 hours after treatment, MZF1 protein was determined using IB assay. **P < 0.01 vs other groups. Results from one representative experiment out of three are shown. Data are represented as means ± SD (n = 6 in vivo, n = 3 in vitro in each group or each time‐point)

In order to explore the potential mechanism of RGC‐32 gene transcription, RGC‐32 gene promoter was analysed. Rat GMCs were transfected with the luciferase reporter plasmids of RGC‐32 gene promoter FL or different deletion fragments, followed by sublytic C5b‐9 incubation for 10 hours. As shown in Figure 2A, sublytic C5b‐9 treatment markedly enhanced the luciferase activity of RGC‐32 gene promoter (FL, −594 ~ +93, −394 ~ +93 and −194 ~ +93 nt), but not +8 ~ +93 nt, suggesting that the −194 ~ +8 nt region might contain some important transcriptional factor‐binding sites. Because the TFsearch software predicted that the −194 ~ +8 nt region could contain the binding elements of some transcriptional factors such as p300, MZF1 and SP‐1 (Figure 2B), two proliferation‐related transcriptional factors of MZF1 and SP‐1 were chosen for further study.^40^, ^41^ Expectedly, the expression of MZF1 and SP‐1 was up‐regulated both in vivo and in vitro; however, only MZF1 exhibited similar expression time‐points with RGC‐32 (Figure 2C‐F), indicating that MZF1 might regulate RGC‐32 gene transcription.

### 3.4 RGC‐32 gene activation is mediated by MZF1 at transcriptional level

**Figure 3:**
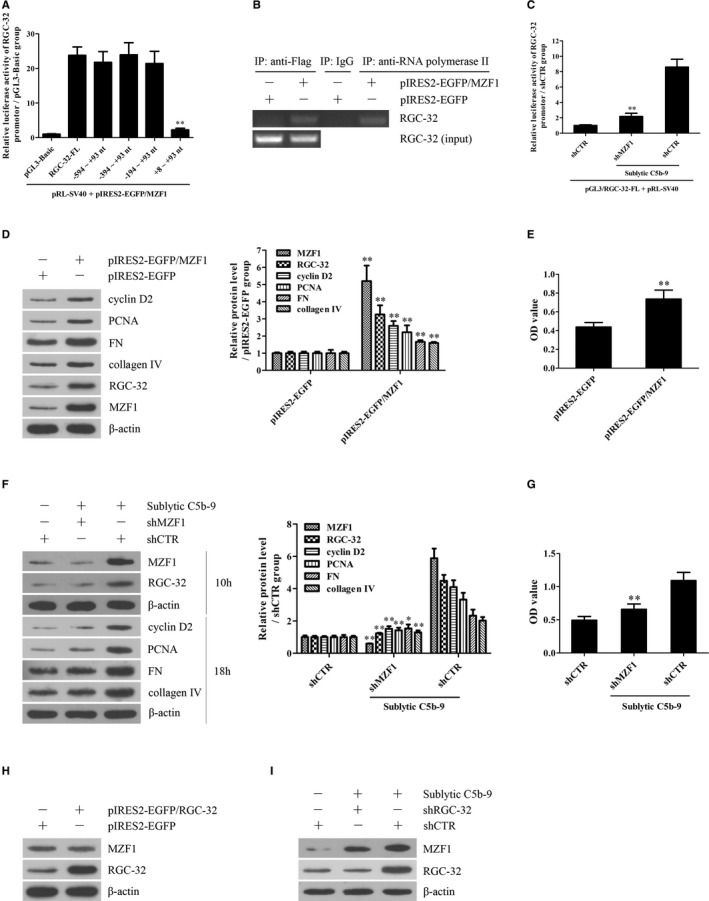
The effects of MZF1 on RGC‐32 gene activation as well as GMC proliferation and ECM production induced by sublytic C5b‐9. A, The plasmids of pIRES2‐EGFP/MZF1 and pGL3/RGC‐32 (FL or different deletion fragments) were co‐transfected into the GMCs and then the luciferase activity was detected at 48 hours after transfection. **P < 0.01 vs FL group. B, The plasmids of pIRES2‐EGFP/MZF1‐Flag and pIRES2‐EGFP were transfected into rat GMCs respectively and then the anti‐Flag antibody or IgG control was used to pull down DNA‐protein complex from the GMCs at 48 hours after transfection. Subsequently, immunoprecipitated DNA was amplified using PCR using a pair of primers for the −220 ~ −50 nt region of RGC‐32 gene promoter. C, The plasmids of pGL3/RGC‐32 (FL), pRL‐SV40 and shMZF1 or shCTR were co‐transfected into rat GMCs for 48 hours followed by sublytic C5b‐9 treatment for 10 hours and then the luciferase activity was detected. **P < 0.01 vs shCTR + sublytic C5b‐9 group. D and E, Rat GMCs were transfected with pIRES2‐EGFP/MZF1 or pIRES2‐EGFP for 48 hours and 72 hours and then the expression levels of MZF1, RGC‐32, cyclin D2, PCNA, FN and collagen IV were determined using IB analysis (D, 48 hours). Meantime, cellular proliferation was detected with CCK‐8 assay (E, 72 h). **P < 0.01 vs pIRES2‐EGFP group. F and G, Rat GMCs were transfected with shMZF1 or shCTR for 48 hours followed by sublytic C5b‐9 stimulation for different time‐points. The expression levels of MZF‐1 and RGC‐32 gene at 10 hours, as well as cyclin D2, PCNA, FN, collagen IV and β‐actin at 18 hours were detected using IP analysis (F). In addition, GMC proliferation at 48 hours was also determined with CCK‐8 assay (G). *P < 0.05, **P < 0.01 vs shCTR + sublytic C5b‐9 group. H and I, Rat GMCs were transfected with pIRES2‐EGFP/RGC‐32, pIRES2‐EGFP, shRGC‐32 or shCTR for 48 h followed by sublytic C5b‐9 stimulation or no stimulation. Then, the expression levels of RGC‐32 and MZF1 were detected using IB assay. Results from one representative experiment out of three are shown. Data are represented as means ± SD (n = 3 in each group for IB, n = 5 in each group for CCK‐8)

Further experiments were performed to evaluate the regulatory effect of MZF1 on RGC‐32 gene promoter activity. First, we observed that exogenous overexpression of MZF1 in the GMCs increased luciferase activity of RGC‐32 gene promoter (FL) and the three deletion fragments (−594 ~ +93, −394 ~ +93 and −194 ~ +93 nt), but had no effect on the shortest deletion fragment (+8 ~ +93 nt), indicating that −194 ~ +8 nt region of RGC‐32 promoter contains an MZF1‐binding site (Figure 3A). A similar result was obtained in 293T cells (Figure S5A). After the predicted MZF1‐binding site (−150 ~ −143 nt) was mutated, the luciferase activity of RGC‐32 gene promoter was markedly decreased (Figure S5B). Next, the ChIP assay demonstrated that the overexpressed MZF1 protein could bind to the −220 ~ −50 nt region of RGC‐32 gene promoter (Figure 3B). Moreover, not only the knockdown of MZF1 but also the mutation of MZF1‐binding element with RGC‐32 promoter remarkably down‐regulated the luciferase activity of RGC‐32 gene promoter (FL) induced by sublytic C5b‐9 (Figure 3C and Figure S6), hinting that sublytic C5b‐9 increases RGC‐32 promoter activity via up‐regulation of MZF1 in the GMCs.

### 3.5 MZF1‐dependent RGC‐32 expression is involved in GMC proliferation and ECM production exposed to sublytic C5b‐9

To determine whether MZF1‐up‐regulated RGC‐32 expression is associated with sublytic C5b‐9‐induced GMC proliferation and ECM production, MZF1 overexpression and knockdown experiments were performed in vitro. As presented in Figure 3D‐G, MZF1 overexpression with pIRES2‐EGFP/MZF1 notably increased RGC‐32, cyclin D2, PCNA, FN and collagen IV expression as well as GMC proliferation (Figure 3D,E), whereas MZF1 knockdown with shMZF1 significantly decreased the above‐mentioned protein expression as well as GMC proliferation stimulated with sublytic C5b‐9 (Figure 3F,G). Furthermore, RGC‐32 overexpression and knockdown had no effect on MZF1 induction in the GMCs induced by sublytic C5b‐9 (Figure 3H,I), confirming that MZF1 is an upstream regulator of RGC‐32 during sublytic C5b‐9‐triggered GMC proliferative response.

### 3.6 ERK5 activation is required for MZF1 and RGC‐32 expression as well as GMC proliferation and ECM secretion incubated with sublytic C5b‐9

**Figure 4:**
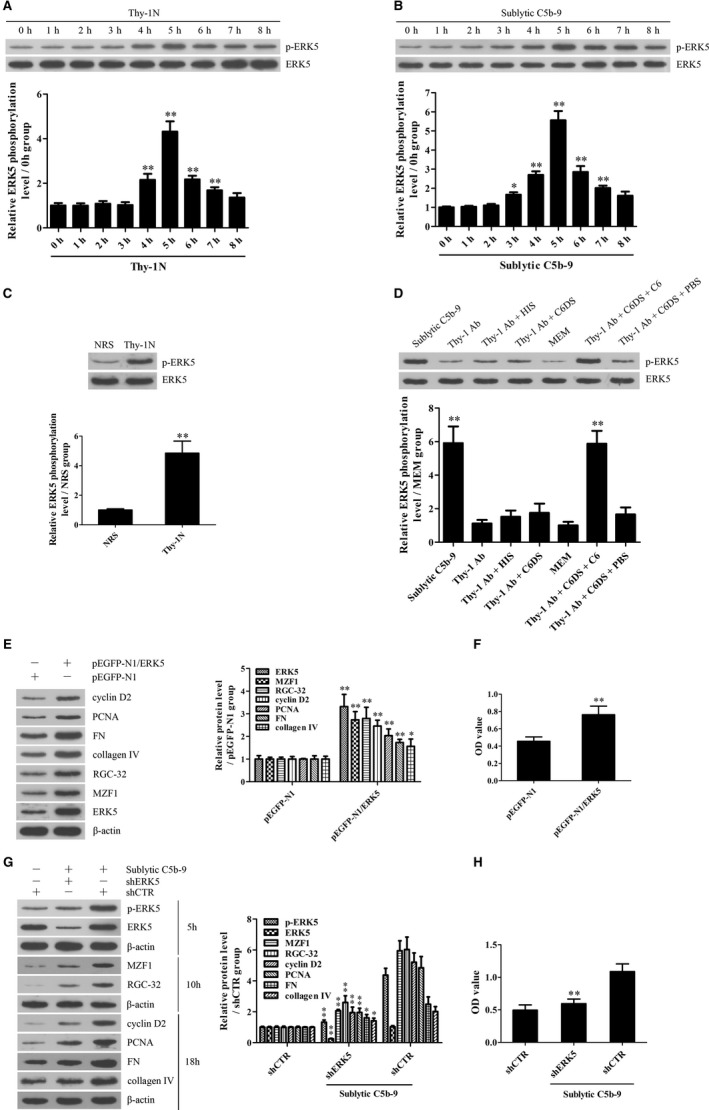
The activation and roles of ERK5 in MZF1 and RGC‐32 expression as well as GMC proliferation and ECM secretion in response to sublytic C5b‐9. A and B, ERK5 phosphorylation in the renal tissues of Thy‐1N rats (A) and in the GMCs exposed to sublytic C5b‐9 (B) was examined using IB assay. *P < 0.05, **P < 0.01 vs 0 hour time‐point. C, ERK5 phosphorylation in the renal tissues of Thy‐1N and NRS rats at 5 hours was examined by IB analysis. **P < 0.01 vs NRS groups. D, Rat GMCs were treated in different groups for 5 hours and then ERK5 phosphorylation was detected using IB. **P < 0.01 vs other groups. E and F, Rat GMCs were transfected with pEGFP‐N1/ERK5 or pEGFP‐N1 for 48 hours and 72 hours and then the expression levels of ERK5, MZF1, RGC‐32, cyclin D2, PCNA, FN and collagen IV were detected using IB (E, 48 hours). Meantime, cellular proliferation was also determined with CCK‐8 assay (F, 72 hours). *P < 0.05, **P < 0.01 vs pEGFP‐N1 group. G and H, Rat GMCs were transfected with shERK5 or shCTR for 48 hours followed by sublytic C5b‐9 treatment for different time‐points. The expression levels of p‐ERK5, t‐ERK5 at 5 hours, MZF‐1 and RGC‐32 at 10 hours, as well as cyclin D2, PCNA, FN and collagen IV at 18 hours were detected using IB (G). Additionally, GMC proliferation at 48 hours was also determined with CCK‐8 assay (H). *P < 0.05, **P < 0.01 vs shCTR + sublytic C5b‐9 group. Results from one representative experiment out of three are shown. Data are represented as means ± SD (n = 6 in vivo, n = 3 in each group for IB in vitro, n = 5 in each group for CCK‐8)

Time course studies revealed that p‐ERK5 increased in a time‐dependent manner and peaked at 5 hours both in vivo and in vitro (Figure 4A‐C). IHC staining further displayed that p‐ERK5 was enhanced mainly in the glomeruli of rats (Figure S7). Grouping experiment in vitro displayed that the p‐ERK5 induction was due to C5b‐9 assembly (Figure 4D). Moreover, overexpression of ERK5 with pEGFP‐N1/ERK5 in the GMCs markedly up‐regulated MZF1, RGC‐32, cyclin D2, PCNA, FN and collagen IV expression as well as cellular proliferation (Figure 4E,F), while knockdown of ERK5 with shERK5 obviously down‐regulated above‐mentioned protein production and GMC proliferation in response to sublytic C5b‐9 (Figure 4G,H), implicating that ERK5 activation is essential for MZF1 and RGC‐32 induction as well as GMC proliferation and ECM production upon sublytic C5b‐9.

### 3.7 FBXO28‐ and TRAF6‐mediated ERK5 activation is necessary for MZF1 and RGC‐32 expression as well as GMC proliferative changes induced by sublytic C5b‐9

**Figure 5:**
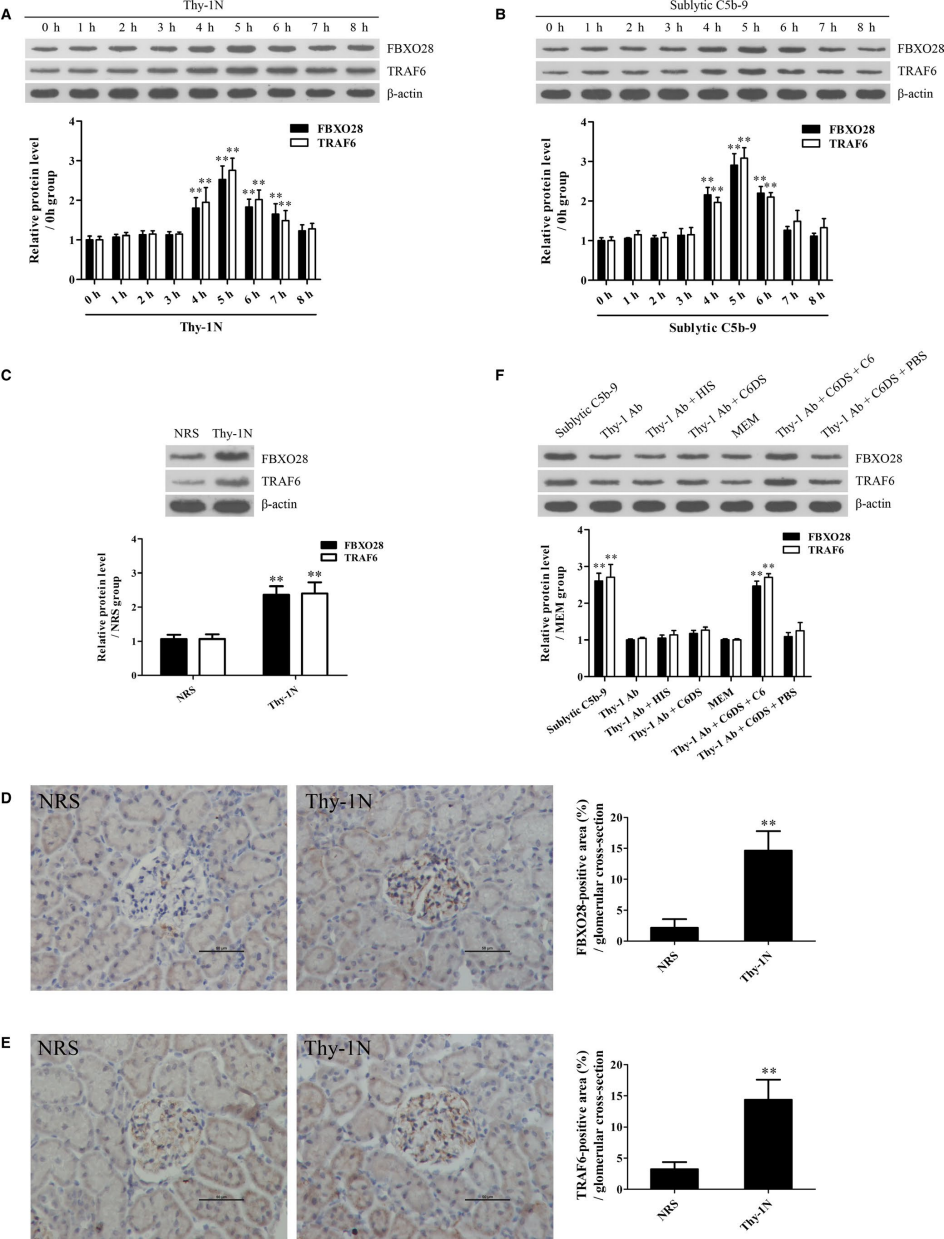
The expression of FBXO28 and TRAF6 in the renal tissues of Thy‐1N rats and in the GMCs stimulated with sublytic C5b‐9. A and B, The protein levels of FBXO28 and TRAF6 in the renal tissues of Thy‐1N rats (A) and in the GMCs exposed to sublytic C5b‐9 (B) for different time‐points were examined using IB assay. *P < 0.05, **P < 0.01 vs 0 hour time‐point. C‐E, The protein levels of FBXO28 and TRAF6 in the renal tissues of Thy‐1N and NRS rats at 5 hours were examined using IB analysis (C) and IHC staining (D and E, Magnification, ×400) respectively. **P < 0.01 vs NRS groups. F, Rat GMCs were divided into different groups. At 5 hours after treatment, the protein levels of FBXO28 and TRAF6 were determined using IB experiments. **P < 0.01 vs other groups. Results from one representative experiment out of three are shown. Data are represented as means ± SD (n = 6 in vivo, n = 3 in vitro in each group or each time‐point)

**Figure 6:**
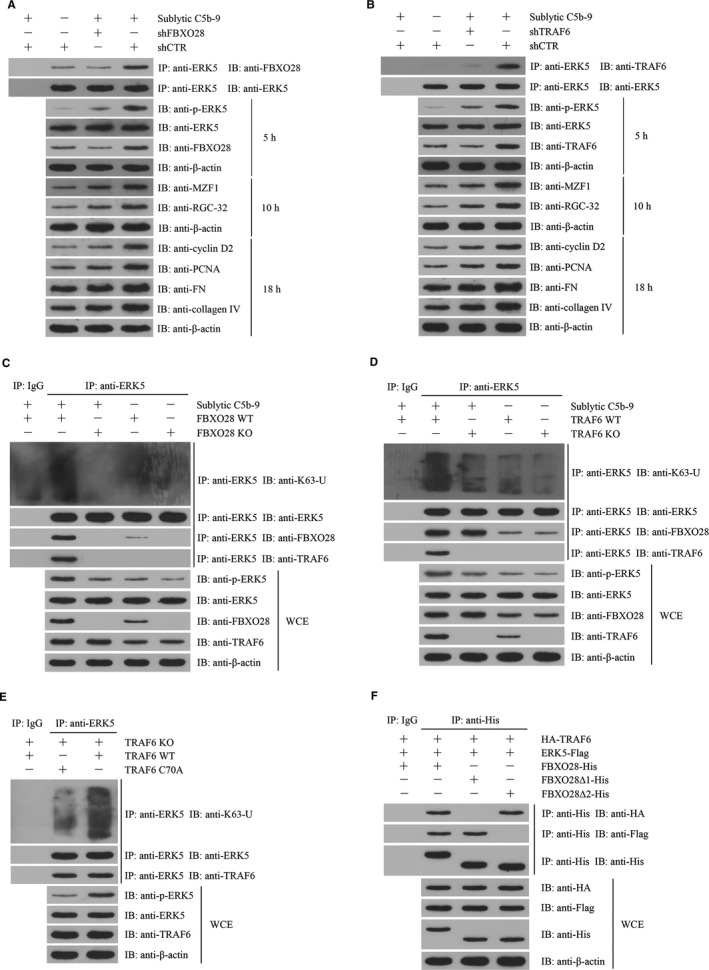
FBXO28‐TRAF6 complex‐mediated ERK5 K63‐ubiquitination is necessary for ERK5/MZF1/RGC‐32 activation as well as GMC proliferative changes in response to sublytic C5b‐9. A and B, Rat GMCs were transfected with shFBXO28 (A) or shTRAF6 (B) for 48 hours followed by sublytic C5b‐9 treatment for different time‐points. The expression levels of FBXO28, TRAF6 and ERk5 at 5 hours, MZF‐1 and RGC‐32 at 10 hours, as well as cyclin D2, PCNA, FN and collagen IV at 18 hours were detected using IB. C and D, Co‐IP experiment was done to pull down ERK5 protein with anti‐ERK5 from FBXO28‐dificient GMCs (C) or TRAF6‐dificient GMCs (D) exposed to sublytic C5b‐9 for 5 hours, the then IB assay was used to detect K63‐ubiquitin, ERK5, FBXO28 and TRAF6 in the co‐IP‐complex as well as FBXO28, TRAF6, ERK5, p‐ERK5, MZF1 and RGC‐32 in WCE. E, TRAF6‐dificient GMCs was transfected with pcDNA3.1/HA‐TRAF6^WT^ or pcDNA3.1/HA‐TRAF6^C70A^ for 48 hours and then the levels of K63‐ubiquitin, ERK5 and HA‐TRAF6 in the co‐IP‐complex and HA‐TRAF6, ERK5 and p‐ERK5 in WCE were detected using IB analysis. F, 293T cells were transfected with pcDNA3.1/FBXO28‐His, pcDNA3.1/FBXO28∆1‐His or pcDNA3.1/FBXO28∆2‐His and pEGFP‐N1/ERK5‐Flag and pcDNA3.1/HA‐TRAF6. Co‐IP experiment was done to pull down FBXO28‐His, FBXO28∆1‐His or FBXO28∆2‐His protein with anti‐His, then IB assay was used to detect ERK5‐Flag, FBXO28‐His and HA‐TRAF6 in the co‐IP‐complex and WCE. Results from one representative experiment out of three are shown. Data are represented as means ± SD (n = 3 in each group)

To find the potential mechanism of ERK5 activation, the ERK5 protein complex was collected from the GMCs stimulated with sublytic C5b‐9 for 5 hours using co‐IP and then the mass spectrometry analysis disclosed that FBXO28 protein was contained in the protein complex (Figure S8). In addition, our previous studies have reported that TRAF6 is involved in Akt1 activation in sublytic C5b‐9‐stimulated GMCs,^11^ therefore we further asked that whether ERK5 activation could also be regulated by FBXO28 and TRAF6. First, we showed that FBXO28 and TRAF6 expression increased in Thy‐1N rat renal tissues and in sublytic C5b‐9‐treated GMCs and peaked at 5 hours with a similar trend of ERK activation (Figure 5A‐F). Then, knockdown of FBXO28 and TRAF6 respectively with shRNA remarkably down‐regulated ERK5 phosphorylation, MZF1, RGC‐32, cyclin D2, PCNA, FN and collagen IV expression and GMC proliferation exposed to sublytic C5b‐9 (Figure 6A,B and Figure S9), indicating that FBXO28‐ and TRAF6‐mediaded ERK5 activation is essential for MZF1 and RGC‐32 expression as well as GMC proliferation and ECM secretion triggered by sublytic C5b‐9.

### 3.8 FBXO28‐TRAF6 complex‐mediated K63‐ubiquitination of ERK5 is necessary for ERK5 activation

In order to identify the possible roles of FBXO28 and TRAF6 in ERK5 ubiquitination and their relationship, FBXO28‐deficient and TRAF6‐deficient rat GMCs were established by CRISPR/Cas9‐mediated gene knockout. We found that sublytic C5b‐9 could markedly enhance ERK5 K63‐ubiquitination and phosphorylation in WT rat GMCs, but not in FBXO28‐ and TRAF6‐deficient rat GMCs (Figure 6C,D), suggesting that both FBXO28 and TRAF6 are necessary for ERK5 K63‐ubiquitination and phosphorylation. While sublytic C5b‐9 treatment exhibited no significant effect on ERK5 K48‐ubiquitination (data not shown). Furthermore, we observed that FBXO28‐deficient GMCs exhibited no protein interaction between TRAF6 and ERK5; however TRAF6‐deficiency did not block the interaction between FBXO28 and ERK5 (Figure 6C,D), implicating that TRAF6 and ERK5 indirectly bound to each other via FBXO28. Additionally, the overexpression of TRAF6 in TRAF6‐deficient GMCs markedly enhanced ERK5 ubiquitination and phosphorylation as well as MZF1 and RGC‐32 expression relative to the overexpression of TRAF6^C70A^ that was deficient in E3 ligase activity (Figure 6E). Collectively, these data indicate that FBXO28 protein can link TRAF6 and ERK5 proteins and promote ERK5 K63‐ubiquitination by TRAF6.

To further find the binding region of FBXO28 for TRAF6 and ERK5 proteins, we constructed deleted FBXO28 including FBXO28∆1 (F‐box domain deletion, 61‐109 aa) and FBXO28∆2 (predicted coiled coil domain deletion, 275‐330 aa).^42^ The expression plasmids of FBXO28, FBXO28∆1 or FBXO28∆2 and the expression plasmids of TRAF6 and ERK5 were co‐transfected into 293T cells. Then, co‐IP experiment revealed that FBXO28∆1 protein could bind to ERK5 but not TRAF6, and differently FBXO28∆2 protein could bind to TRAF6 rather than ERK5 (Figure 6F), confirming that F‐box domain of FBXO28 is essential for TRAF6‐binding, and predicted coiled coil domain of FBXO28 is required for ERK5‐binding.

### 3.9 Knockdown of renal FBXO28, TRAF6, ERK5, MZF1 and RGC‐32 genes abolishes GMC proliferation, ECM accumulation and urinary protein secretion of Thy‐1N rats

**Figure 7:**
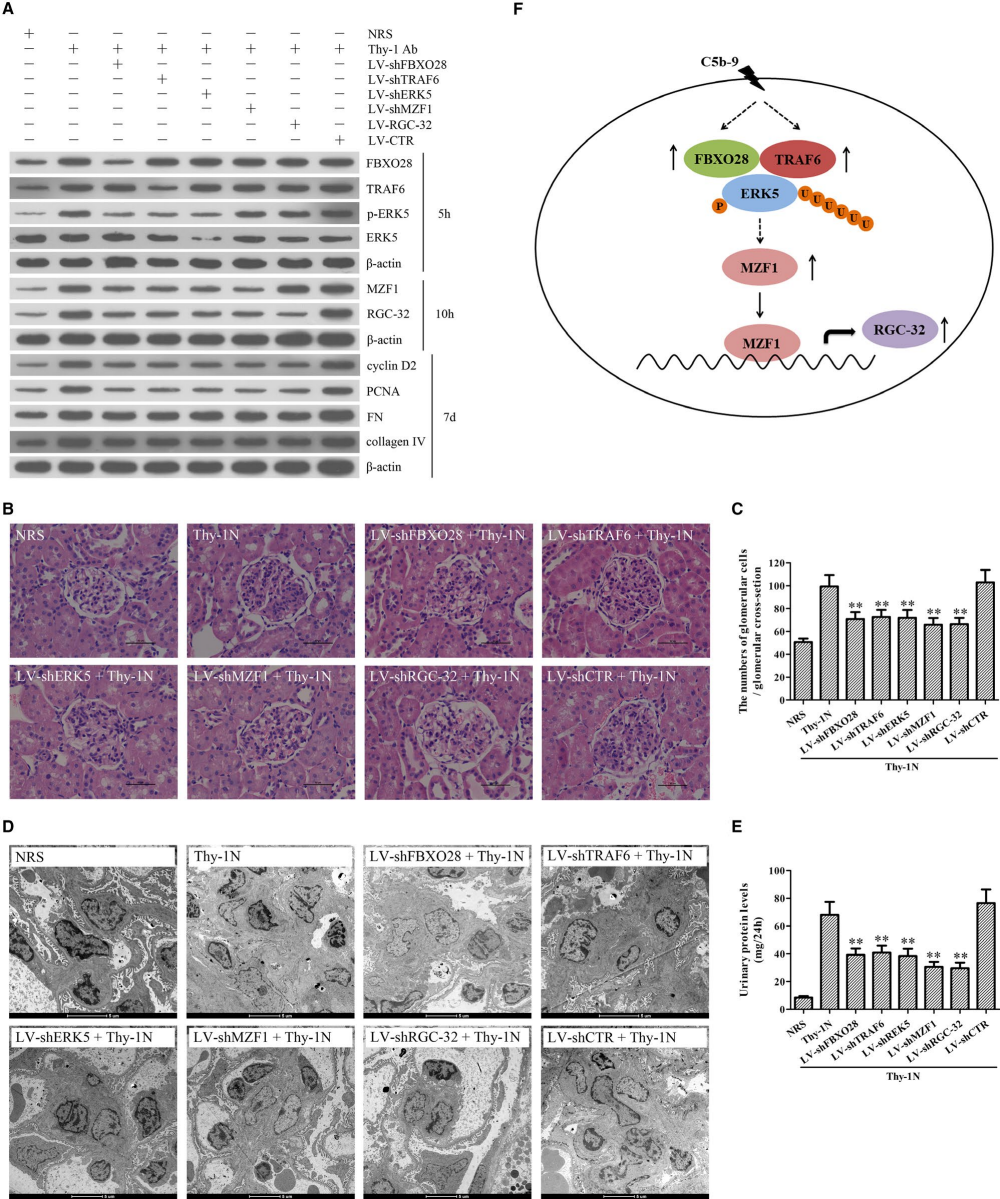
Knockdown of renal FBXO28, TRAF6, ERK5, MZF1 and RGC‐32 gene abolished GMC proliferation and ECM secretion in Thy‐1N rats. LV‐shFBXO28, LV‐shTRAF6, LV‐shERK5, LV‐shMZF1 and LV‐shRGC‐32 were used to silence target genes, and then Thy‐1N was induced 4 days later. A, The protein levels of FBXO28, TRAF6, p‐ERK5, t‐ERK5, MZF1, RGC‐32, cyclin D2, PCNA, FN and collagen IV in the renal tissues of rats were detected using IB assay (at 5 hours for FBXO28, TRAF6, p‐ERK5 and t‐ERK5, at 10 hours for MZF1 and RGC‐32, on 7 d for cyclin D2, PCNA, FN and collagen IV). B and C, The numbers of total glomerular cells in different groups of rats on 7 d after Thy‐1N induction was observed using Haematoxylin and eosin under LM (Magnification, ×400). D, GMC proliferation and ECM accumulation in different groups of rats on 7 d after Thy‐1N induction were evaluated by EM. E, The content of urinary protein (mg/24 h) of rats on day 7 after Thy‐1N induction was detected. F, A schematic drawing for the pathway we revealed. **P < 0.01 vs Thy‐1N group and LV‐shCTR + Thy‐1N group. Results from one representative experiment out of three are shown. Data are represented as means ± SD (n = 6 in each group)

To further demonstrate the roles of these genes in rat Thy‐1N, the LV‐shFBXO28, LV‐shTRAF6, LV‐shERK5, LV‐shMZF1 and LV‐shRGC‐32 were used to silence corresponding target genes in the renal tissues of rats, and then Thy‐1N was induced 4 days later. We found that (a) Knockdown of FBXO28 and TRAF6 decreased ERK5 phosphorylation as well as MZF1 and RGC‐32 expression. (b) Knockdown of ERK5 reduced MZF1 and RGC‐32 expression, but had no effect on FBXO28 and TRAF6 expression. (c) Knockdown of MZF1 decreased RGC‐32 expression, but had no effect on FBXO28 and TRAF6 expression as well as ERK5 phosphorylation. (d) Knockdown of RGC‐32 had no effect on others. Furthermore, after the expression of the above‐mentioned genes was effectively inhibited, the expression levels of renal cyclin D2, PCNA, FN and collagen IV were all significantly down‐regulated (Figure 7A). Meanwhile, knockdown of FBXO28, TRAF6, ERK5, MZF1 and RGC‐32 genes reduced the number of total glomerular cells in Thy‐1N rats (Haematoxylin and eosin staining, Figure 7B,C) as well as GMC proliferation and ECM accumulation (EM, Figure 7D). Besides, knockdown of the above‐mentioned genes could also lessen the content of urinary protein (mg/24 h) of Thy‐1N rats on day 7 (Figure 7E).

## 4 DISCUSSION

As a proliferation‐related gene, RGC‐32 expression can be induced by sublytic C5b‐9 in endothelial cells and smooth muscle cells and contributes to the proliferation of these cells.^15^, ^43^, ^44^ For example, Vlaicu et al have reported that RGC‐32 expression is increased in the human aortic atherosclerotic wall and related with C5b‐9 deposition and atherosclerosis progression. Silencing of RGC‐32 expression abolishes C5b‐9‐induced proliferation of human aortic endothelial cells in vitro.^15^ Reportedly, RGC‐32 expression is also involved in TGF‐β1‐induced ECM production from astrocytes of experimental autoimmune encephalomyelitis (EAE) mice.^44^ However, the expression and role of RGC‐32 in the GMC proliferative response triggered by sublytic C5b‐9 in rat Thy‐1N remains unclear. Our present studies showed that RGC‐32 expression was markedly elevated in the renal tissues of Thy‐1N rats (in vivo) and in the GMCs exposed to sublytic C5b‐9 (in vitro). Functional experiments in vitro showed that RGC‐32 up‐regulation was essential for not only GMC proliferation but also ECM secretion in response to sublytic C5b‐9, indicating that RGC‐32 plays important roles in sublytic C5b‐9‐induced GMC proliferative changes.

Promoter truncation experiment exhibited that the −194 ~ +8 nt region of RGC‐32 gene promoter was important for its transcription in sublytic C5b‐9‐stimulated GMCs. The TFsearch software predicted that the above‐mentioned promoter region could contain the binding elements of two proliferation‐related transcriptional factors (MZF1 and SP‐1). While only MZF1 displayed the same expression time‐points with RGC‐32 both in vivo and in vitro. Further the ChIP and promoter mutation assay disclosed that MZF1 could bind to the −150 ~ −143 nt region of RGC‐32 gene promoter. Functionally, MZF1 up‐regulation was involved in RGC‐32 expression as well as GMC proliferation and ECM production exposed to sublytic C5b‐9 stimulation in vitro. Notably, there is no report on MZF1‐regulated RGC‐32 gene transcription and expression in other cells before we reveal the role of MZF1 in the regulation of RGC‐32 gene expression as a transcription factor. Reportedly, MZF‐1 could promote the proliferation of some cell types such as NIH3T3 fibroblasts and IL‐3‐dependent multipotent hematopoietic FDCP.1 cells;^41^, ^45^ however, several researchers also pointed out that MZF‐1 suppressed the proliferation of prostate cancer cells and colon cancer cells,^46^, ^47^ implying that MZF1 is a multifunctional protein and may play different roles in different cell types.

As for the upstream signalling molecule of MZF1‐RGC‐32‐mediated GMC proliferative changes, we found that p‐ERK5 was increased both in vivo and in vitro. Moreover, overexpression and knockdown experiments demonstrated that ERK5 activation was required for MZF1 and RGC‐32 expression as well as GMC proliferation and ECM production induced by sublytic C5b‐9. Our present studies further revealed that FBXO28 and TRAF6 expression was increased in vivo and in vitro. FBXO28 and TRAF6 could form a protein complex and bind to ERK5 protein, in the which F‐box domain and the predicted coiled coil domain of FBXO28 were involved in TRAF6‐ and ERK5‐binding respectively. Finally, the interaction of FBXO28, TRAF6 and ERK5 proteins promoted ERK5 K63‐ubiquitination and phosphorylation as well as MZF1 and RGC‐32 expression in rat GMCs. Reportedly, F‐box proteins are critical components of the SCF type E3 ubiquitin ligase complex. Our findings revealed that FBXO28 protein as a member of the FBXO family could link TRAF6 and ERK5 proteins via two different domains within FBXO28 (ie F‐box domain and predicted coiled coil domain) and induce the K63‐ubiquitination of ERK5 mediated by TRAF6.

As for the mechanism of K63‐ubiquitination‐regulated ERK5 phosphorylation, reportedly the K63‐ubiquitination of target proteins increases their binding and phosphorylation by other kinases or promoting their homodimer formation and trans‐autophosphorylation. For example, Zhang et al^48^ have displayed that Akt K63‐ubiquitination enhances the interaction of cytoplasmic Akt and membrane‐bound phosphatidylinositol 3,4,5‐trisphosphate (PIP3) in mouse macrophages and subsequently increases Akt phosphorylation by PIP3‐bound 3‐phosphoinositide‐dependent protein kinase (PDK). Moreover, Pourcelot et al^49^ have also found that the ubiquitin‐binding protein optineurin and K63‐ubiquitinated TANK‐binding kinase 1 (TBK1) can form protein complex in the Golgi apparatus of murine embryonic fibroblasts (MEFs) and 293T cells after the stimulation of RIG‐I‐like receptors (RLRs) or Toll‐like receptor‐3 (TLR3) and promote TBK1 homodimer formation and TBK1 trans‐autophosphorylation. Here, although our experiments manifested that FBXO28 could link TRAF6 and ERK5 and then promote ERK5 K63‐ubiquitination and phosphorylaton, the precise mechanism about the K63‐ubiquitination‐induced ERK5 phosphorylation in sublytic C5b‐9‐stimulated GMCs still needs to be clarified in the future.

In addition to the above‐mentioned in vitro studies, our in vivo studies also revealed that silencing of renal FBXO28, TRAF6, ERK5, MZF1 and RGC‐32 genes inhibited GMC proliferation and ECM accumulation as well as urinary protein production of Thy‐1N rats. Collectively, these findings suggest that sublytic C5b‐9 might induce the proliferation of GMCs in Thy‐1N rats through the activation of ERK5/MZF1/RGC‐32 axis mediated by the FBXO28‐TRAF6 complex (Figure 7F) and these might provide a new insight into the pathogenesis of MsPGN.

## CONFLICT OF INTEREST

All the authors declared no competing interests.

## AUTHOR CONTRIBUTIONS

WQ and YWW conceived and designed the study. WQ supervised all experiments. TYY, LLW, CHZ, BMQ, CLY, FXH, YFZ, MYC, ML, DZ, and JZ performed all experiments. TYY, LLW, CHZ, BMQ and WQ analysed the data. WQ and YWW wrote the manuscript. All authors read and discussed the manuscript.
