# Supplementary material for: Sublytic C5b‐9 induces proliferation of glomerular mesangial cells via ERK5/MZF1/RGC‐32 axis activated by FBXO28‐TRAF6 complex
Source: J Cell Mol Med. 2019 Jun 11;23(8):5654–71. doi: 10.1111/jcmm.14473 (PMC6653533; doi:10.1111/jcmm.14473)
Supplement: Supplementary file 1 [file JCMM-23-5654-s001.docx]

**Table S1**

|  | forward primer (5’→3’) | reverse primer (5’→3’) |
| --- | --- | --- |
| ERK5 | CCGCTCGAGAGCCCTCGGGACGATCTTC | CGGGATCCTTAATGGTGATGGTGATGATGGGGTTCTTGGAGGTCAGGC |
| MZF1 | GAAGATCTTCAGGGACAGACACTGGCCTCAAA | ACGCGTCGACTTACTTATCGTCGTCATCCTTGTAATCCTCAGTGCTGTGGATGCGTTGA |
| RGC-32 | GAAGATCTTCAACACCTCTCAGCATCTCTACTACA | ACGCGTCGACTTAATGGTGATGGTGATGATGCATACTTGCTAAGGTTCTGTCCAG |

**Table S1** Primers for construction of ERK5, MZF1 and RGC-32 expression plasmids.

Three pairs of primers were designed to amplify rat ERK5, MZF1 and RGC-32 gene CDS region respectively. The sequence of these primers was shown. Restriction enzyme sites were underlined (ERK5 upstream: *Xho* I; ERK5 downstream: *BamH* I; MZF1 upstream: *Bgl* II; MZF1 downstream: *Sal* I; RGC-32 upstream: *Bgl* II; RGC-32 downstream: *Sal* I). His-tag was added to the 3’ of ERK5 and RGC-32 gene CDS by PCR. Flag-tag was added to the 3’ of MZF1 gene CDS by PCR. The sequence encoding His and Flag was labeled with double underline.

**Table S2**

|  | forward primer (5’→3’) | reverse primer (5’→3’) |
| --- | --- | --- |
| -1094 ~ +93 nt | CGACGCGTGTCACAGGTGAATGTTGTGCATG | GAAGATCTGGAGAGGTTGCTCAGTGACACG |
| -594 ~ +93 nt | CGACGCGTATGGCCCTATACCGGAATGG | GAAGATCTGGAGAGGTTGCTCAGTGACACG |
| -394 ~ +93 nt | CGACGCGTGCAAGCAAGGAGGCTCTGC | GAAGATCTGGAGAGGTTGCTCAGTGACACG |
| -194 ~ +93 nt | CGACGCGTTCTCCGGAGGCCGGCG | GAAGATCTGGAGAGGTTGCTCAGTGACACG |
| +8 ~ +93 nt | CGACGCGTACCCGAGCGGACCGC | GAAGATCTGGAGAGGTTGCTCAGTGACACG |

**Table S2** Primers for full-length and different deletion fragments of RGC-32 gene promoter.

Five pairs of primers were designed to amplify full-length (-1094 ~ +93 nt) and four different deletion fragments (-594 ~ +93, -394 ~ +93, -194 ~ +93 and +8 ~ +93 nt) of RGC-32 gene promotor. The sequence of these primers was shown. Restriction enzyme sites were underlined (upstream: *Mlu* I; downstream: *Bgl* II).

**Figure S1**


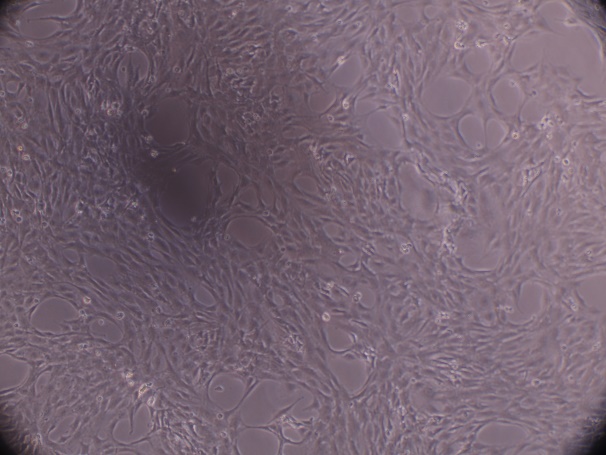

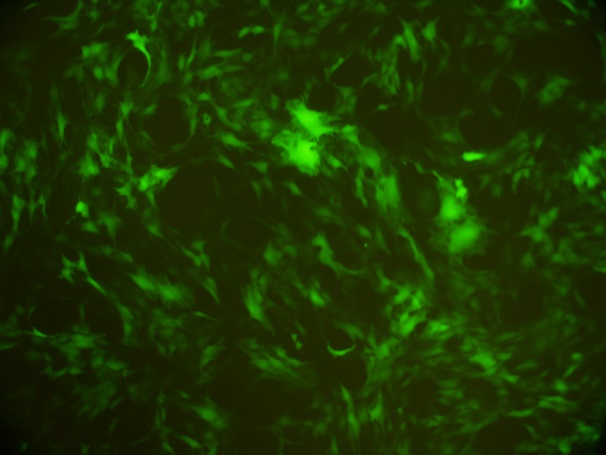


**Figure S1** Transfection efficiency of shRNA expression plasmids into rat GMCs.

Transient transfection of shCTR expression plasmids into the cultured GMCs was conducted with Neon^TM^ transfection system according to the manufacturer's procedure. The transfection efficiency of shRNA expression plasmids was detected by the fluorescence of GFP at 48 h after transfection (right), and meanwhile, the same visual field were observed under ordinary light (left). The transfection efficiency of plasmids was 80% ~ 90% by counting **the** [**ratio**](http://www.iciba.com/ratio/) [**of**](http://www.iciba.com/of/) **GFP expressing cells to total cells**. The representative fluorescence microscopic images have been displayed (Original magnification: ×100).

**Figure S2**

**A**


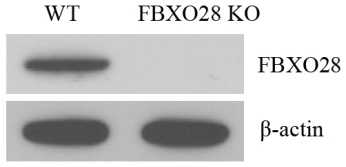


**B**


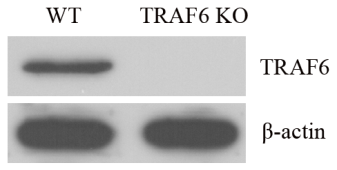


**Figure S2** Identification of CRISPR/Cas9-mediated FBXO28 or TRAF6 gene knockout in rat GMCs.

FBXO28-deficient or TRAF6-deficient rat GMCs cell lines were established by CRISPR/Cas9. FBXO28 gene knockout or TRAF6 gene knockout was identified by IB analysis, and no expression of FBXO28 (A) or TRAF6 (B) was observed in FBXO28- or TRAF6-deficient rat GMCs.

**Figure S3**

Control Lv-shCTR


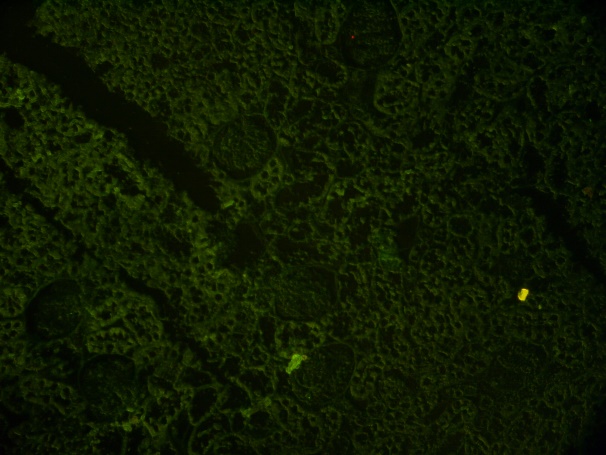

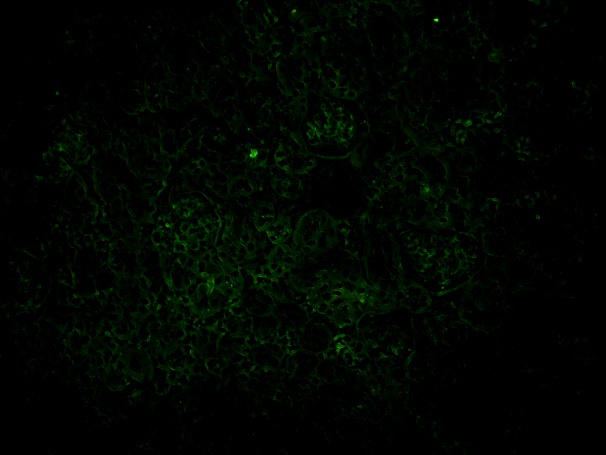


**Figure S3** Transfection efficiency of LV-shRNA *in vivo*.

The LV-shCTR was transfected into rat kidneys via renal artery perfusion suddenly followed by renal veins occlusion for 10 minutes. The protein of GFP in glomeruli was directly observed under a fluorescence microscope to evaluate transfection efficiency. At 96 h after transfection, the expression of GFP was extensively observed in glomeruli and renal tubules. But, no significant GFP expression was found in the renal tissues of rats without transfection. The result demonstrated that LV-shRNA could be effectively delivered to glomeruli by renal artery perfusion followed by renal veins occlusion for 10 minutes. Here, the representative pictures were given (Frozen sections, Original magnification: ×100).

**Figure S4**


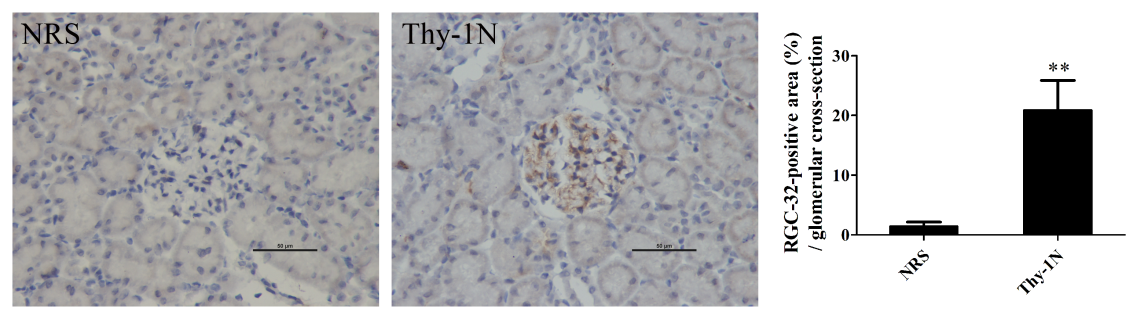


**Figure S4** RGC-32 expression in the renal tissues of rats.

The protein levels of RGC-32 in the renal tissues of Thy-1N and NRS rats at 10 h were examined by IHC staining (Magnification, ×400). ** *P*<0.01 vs. NRS group. Results from one representative experiment out of three were shown. Data were represented as means ± SD (n=6 in each group).

**Figure S5**

**A**

**

**

**B**

**

**

**Figure S5** Effects of MZF1 on RGC-32 promotor activity.

A, The plasmids of pIRES2-EGFP/MZF1 and pGL3/RGC-32 (FL, -594 ~ +93, -394 ~ +93, -194 ~ +93 or +8 ~ +93 nt) were co-transfected into the 293T cells, and then the luciferase activity in the cells was detected at 48 h after transfection. ** *P*<0.01 vs. FL group. B, pIRES2-EGFP/MZF1 and pGL3/RGC-32-FL-WT or pGL3/RGC-32-FL-M were co-transfected into the 293T cells, and then the luciferase activity in the cells was detected at 48 h after transfection. ** *P*<0.01 vs. FL-WT group. Results from one representative experiment out of three were shown. Data were represented as means ± SD (n=3 in each group).

**Figure S6**





**Figure S6** Effects of MZF1-binding element mutation on RGC-32 promotor activity in GMCs in response to sublytic C5b-9.

The plasmids of pGL3/RGC-32-FL-WT and pGL3/RGC-32-FL-M were transfected into the rat GMCs followed by sublytic C5b-9 stimulation for 10 h, and then the luciferase activity in the cells was detected. ** *P*<0.01 vs. pGL3/RGC-32-FL-WT group. Results from one representative experiment out of three were shown. Data were represented as means ± SD (n=3 in each group).

**Figure S7**

**
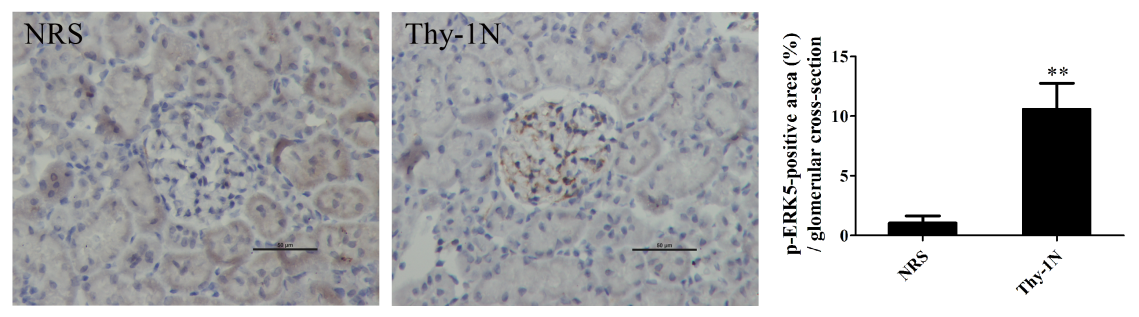
**

**Figure S7** ERK5 phosphorylation in the renal tissues of rats.

The phosphorylation levels of ERK5 in the renal tissues of Thy-1N and NRS rats at 5 h were examined by IHC (Magnification, ×400) staining. ** *P*<0.01 vs. NRS groups. Results from one representative experiment out of three were shown. Data were represented as means ± SD (n=6 in each group).

**Figure S8**


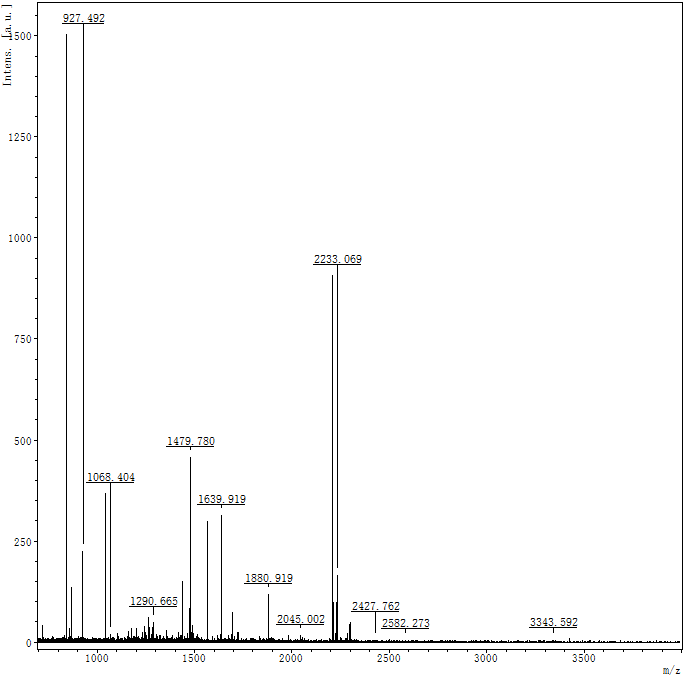


**Figure S8** The identification of FBXO28 by spectrum.

Anti-ERK5 antibody was used to perform co-IP to pull down ERK5 protein complex from the GMCs stimulated with sublytic C5b-9 for 5 h, and then 50 µg of protein was run in a SDS-PAGE gel. Subsequently, the target protein was obtained again by cutting the gel after silver staining. Finally, mass spectrometry analysis was performed and FBXO28 was found in ERK5 protein complex.

**Figure S9**

**A**

**

**

**B**

**

**

**Figure S9** The effects of shFBXO28 and shTRAF6 on GMC proliferation induced by sublytic C5b-9.

Rat GMCs were transfected with shFBXO28 (A) or shTRAF6 (B) for 48 h followed by sublytic C5b-9 treatment for 48 h. Then, GMC proliferation was determined with CCK-8 assay. ** *P*<0.01 vs. shCTR + sublytic C5b-9 group. Results from one representative experiment out of three were shown. Data were represented as means ± SD (n=5 in each group).
